# Chemosensory Event-Related Potentials in Response to Nasal Propylene Glycol Stimulation

**DOI:** 10.3389/fnhum.2019.00099

**Published:** 2019-03-20

**Authors:** Mohammad Sirous, Nico Sinning, Till R. Schneider, Uwe Friese, Jürgen Lorenz, Andreas K. Engel

**Affiliations:** ^1^Department of Neurophysiology and Pathophysiology, University Medical Center Hamburg-Eppendorf, Hamburg, Germany; ^2^Faculty of Life Science, MSH Medical School Hamburg, Hamburg, Germany; ^3^Faculty of Life Science, Laboratory of Human Biology and Physiology, Applied Science University, Hamburg, Germany

**Keywords:** olfaction, EEG, chemosensory response, trigeminus, olfactometer, propylene glycol, 1.2 propanediol

## Abstract

Propylene glycol, also denoted as 1.2 propanediol (C_3_H_8_O_2_), often serves as a solvent for dilution of olfactory stimuli. It is supposed to serve as a neutral substance and has been used in many behavioral and electrophysiological studies to dilute pure olfactory stimuli. However, the effect of propylene glycol on perception and on neuronal responses has hitherto never been studied. In this study we tested by means of a threshold test, whether a nasal propylene glycol stimulation is recognizable by humans. Participants were able to recognize propylene glycol at a threshold of 42% concentration and reported a slight cooling effect. In addition to the threshold test, we recorded electroencephalography (EEG) during nasal propylene glycol stimulation to study the neuronal processing of the stimulus. We used a flow olfactometer and stimulated 15 volunteers with three different concentrations of propylene glycol (40 trials each) and water as a control condition (40 trials). To evaluate the neuronal response, we analyzed the event-related potentials (ERPs) and power modulations. The task of the volunteers was to identify a change (olfactory, thermal, or tactile) in the continuous air flow generated by the flow olfactometer. The analysis of the ERPs showed that propylene glycol generates a clear P2 component, which was also visible in the frequency domain as an evoked power response in the theta-band. The source analysis of the P2 revealed a widespread involvement of brain regions, including the postcentral gyrus, the insula and adjacent operculum, the thalamus, and the cerebellum. Thus, it is possible that trigeminal stimulation can at least partly account for sensations and brain responses elicited by propylene glycol. Based on these results, we conclude that the use of high propylene glycol concentrations to dilute fragrances complicates the interpretation of presumed purely olfactory effects.

## Introduction

The human nasal mucosa contains a variety of sensory receptors and nerve endings enabling it not only to respond to odors, but also to tactile, thermal, and noxious stimuli. These additional sensations arise from activation of the trigeminal nerve, which divides into three main branches two of which innervate the nasal cavity. Branches of the ophthalmic nerve extend into the upper region of the nasal cavity and the maxillary nerve reaches to the lower region of the nasal cavity (Tubbs et al., 2015).

The development of tangible flow-olfactometers enabled the controlled stimulation of the nasal cavity time-locked to electroencephalography (EEG) recordings and opened a way to study the neural processing of chemical stimulation of the olfactory and trigeminal senses using the methodology of (ERPs; Kobal and Plattig, 1978). By embedding the volatile odorant into the continuous airstream, the device prevents a pressure change related to the stimulus, which would lead to a tactile sensation.

The temporally precise stimulation of the olfactory receptors without additional activation of the intranasal trigeminal branching and vice versa offered new possibilities for the separate electrophysiological examination of these two systems. Olfactory ERPs, for example, can be used to perform an objective smell test (Hummel et al., 2000), that is, a measurement that assesses olfactory perception based on electrophysiological data and not on subjective responses. The consideration of neuronal low-frequency oscillatory activities (<30 Hz) also showed clear differences between trigeminal and olfactory stimulation and can be used as a method for clinical evaluation of olfactory perception (Huart et al., 2012, 2013). Source analyses of ERPs showed that the neuronal responses in both modalities clearly differ at early stages of processing (<300 ms) but subsequently resemble each other (Iannilli et al., 2013).

All methods mentioned require a precise control of the stimulus concentration, which exceeds the stimulus threshold, but does not activate additional areas due to high stimulation. The stimulus concentration can be controlled by regulating the airflow of the flow-olfactometer or by diluting the substances. The dilution is often achieved by the addition of propylene glycol (C_3_H_8_O_2_). However, the potential effects of propylene glycol on the trigeminal or olfactory system is not well studied in humans. Nevertheless, pure olfactory stimulants are often mixed with propylene glycol and conclusions are drawn from the supposedly pure olfactory stimulation (Gottfried and Dolan, 2003; Zhou et al., 2010; Kollndorfer et al., 2015). In previous EEG and MEG studies using olfactory stimulation without propylene glycol dilution early activation of primary olfactory cortex was found (Lascano et al., 2010; Stadlbauer et al., 2016).

In this study, we investigated whether propylene glycol is a neutral substance and can be perceived by human participants. In addition, we analyzed the neural responses to propylene glycol stimulation by means of EEG. To this end, participants were stimulated with three different concentrations of propylene glycol while electroencephalographic data was recorded with high spatial density (128 channel EEG). Detection thresholds were obtained for different levels of propylene glycol concentrations. Chemosensory ERPs (CSERPs) and time-frequency analyses were computed. In addition, the sources of electrophysiological activity were located using exact low-resolution electromagnetic tomography (eLORETA).

## Materials and Methods

### Participants

Nineteen healthy, right-handed volunteers (11 females, eight males) with an average age of 23.7 (± 3.5) years participated in the experiment and were paid 10 €/h for their participation. Four participants were excluded from further analysis due to a low detection rate (*d*’-values for strongest condition <1). This study was carried out in accordance with the recommendations of Hamburg Medical Association (Ärztekammer Hamburg) with written informed consent from all subjects. All subjects gave written informed consent in accordance with the Declaration of Helsinki. The protocol was approved by the Hamburg Medical Association (Ärztekammer Hamburg). All participants signed a document of informed consent and completed a questionnaire to rule out any smell dysfunction. Participants reported no history of neurological or psychiatric illness and confirmed to be non-smokers. Normal olfactory function was ensured using the Sniffin’ Sticks test. Participants were instructed to refrain from eating and drinking of stimulants (e.g., coffee and spicy food) or the use of perfumed soaps prior to the experiment.

### Stimuli

The chemosensory stimulus was propylene glycol at low (25%), medium (50%) and high concentration (100%). All dilutions were made by flow-dilution (a neutral dilution airstream is passed to the airstream containing the fragrance). Propylene glycol is an alkanol, which is often used for dilution of odorants. As a control condition distilled water was used as a neutral stimulus. The control stimulus was applied with the same percentage of humidification as the chemosensory stimulus (>60%). The stimuli were delivered to the participants using a Burghart OL023 OM6 olfactometer (Burghart Messtechnik, Wedel, Germany) with a continuous airflow of 8 l/min. The airflow was warmed up to body temperature and conditioned to >60% relative humidity. The control stream needs to be humidified to ensure that the continuous application of air does not dehydrate the mucosa. All participants were stimulated through the right nostril. The stimulus duration was 200 ms and the inter-stimulus interval (ISI) was on average 35.3 (± 8.5) s. Flow time measurements of propylene glycol in the olfactometer were performed by means of a breathing sensor (OL014, Burghart Messtechnik) to ensure stimulation without latencies and pressure changes. White noise of approximately 80 dB was presented via headphones to mask switching clicks of the stimulator.

### Procedure

Participants were seated in an acoustically and electrically shielded chamber in front of a computer monitor with a refresh rate of 100 Hz at a viewing distance of 50 cm. The measurement was divided into four parts. In the first part, every participant underwent a validated odor identification and odor threshold Sniffin’ Sticks test to ensure that the participants were normosmic and that their sense of smell was appropriate (Hummel et al., 2007).

In the second part, the participants’ breathing cycle was determined. Since the processing of olfactory and trigeminal information is related to the breathing cycle, it was ensured that all participants were stimulated at the same time point (200 ms after the inhalation onset; Gudziol and Wächter, 2004). This required to measure and control the participant’s respiration phase. Therefore, a differential pressure transducer was used to record the respiratory oral pressure changes. The participants were trained to breathe orally and to use the technique of velopharyngeal closure (closure of the nasal airway by the elevation of the soft palate). A custom made Matlab application enabled the evaluation of the participants tidal breathing pattern.

**Figure 1:**
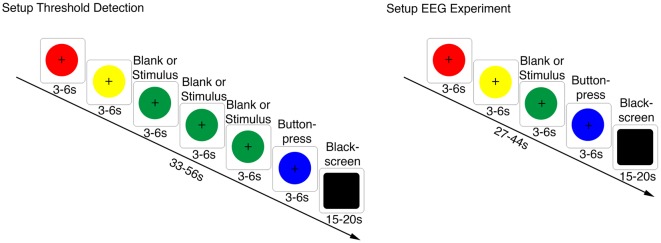
Trial structure for the threshold test and the subsequent electroencephalography (EEG) measurement. Left: setup for the threshold test (three alternative forced choice). Each circle visualized a respiratory cycle, which was recorded previously. The radius of the circle guided the respiration (radius increase = inhalation; radius decrease = exhalation; constant radius = inspiratory hold). The first two circles (red, yellow) led the participant into the tidal breathing rhythm. The airstream contained randomly propylene glycol during the visualization of one of the following three green circles. The remaining two served as blanks. In the presence of these circles the participant was stimulated with distilled water. The blue circle served as a cue for the button response followed by a black screen. Right: setup for the main experiment including the EEG measurement. The green circle contained randomly distilled water or propylene glycol in different concentrations (25%, 50% or 100%). Participants had to indicate, whether the continuous airstream had changed during the green circle by a button press (during presentation of the blue circle).

In the third part, the flow olfactometer was used to perform a detection threshold test with propylene glycol. The thresholds were obtained with a single-staircase, three-alternative forced-choice procedure (Hummel et al., 2007). The previously recorded breathing cycle was visualized as a circle with increasing and decreasing radius on the monitor and the participants were instructed to coordinate their respiration with the circle radius (inhalation during radius increase; exhalation during radius decrease; inspiratory hold during a constant radius = plateau pressure level). Six circles following up each other were presented, each circle leading one respiratory cycle. The order of the colors of the circles was always red, yellow, green, green, green, blue. The first two circles (colored red and yellow) guided the participant into the tidal breathing rhythm. The following three circles (all colored in green) represented the three alternatives in the forced-choice paradigm. The propylene glycol stimulus was randomly administered with one of the three green circles (water with the other two). The participants were instructed to compare the nasal stimulation during the presentation of the three green circles and identify the one different from the other two. During the last circle (colored in blue), the participants had to indicate the number of the green circle with a button press of their right index finger. They were instructed to respond as fast as possible. Each trial ended by presentation of a black screen for 15–20 s to prevent habituation to the stimulus and to enable relaxation of the participants. The stimulus duration (propylene glycol and distilled water) was set to 200 ms. All stimuli were presented 200 ms after inhalation onset. The ISI depended on the duration of the individual breathing cycle and lasted on average 44.3 (± 11.5) s (Figure 1). The stimulus range was from 6.25 to 100% propylene glycol in nine steps (6.25%, 12.5%, 25%, 37.5%, 50%, 62.5%, 75%, 87.5%, 100%). The first stimulus contained 6.25% propylene glycol. Correct detection of the circle containing the substance in two consecutive trials led to a decrease of the propylene glycol concentration by one step in the upcoming trial. One incorrect answer on the other hand, led to an increase of the stimulus concentration by one step. The measurement ended after twelve turning points (either from ascending to descending concentration or vice versa).

In the fourth part, the main experiment was conducted. The neural responses were recorded by means of EEG. Participants were instructed to maintain visual fixation to a fixation cross positioned in the center of the monitor during the trials to avoid blinking and saccade artifacts. The main experiment was a modification of the threshold-test in the third part. It included the presentation of four instead of six circles. The first two circles (red and yellow) guided the participant into the tidal respiration cycle and the third contained randomly propylene glycol in high (100%), medium (50%), low (25%) or zero (distilled water) concentration. The participants were instructed to detect any kind of nasal sensation (olfactory, thermal, or tactile) besides the constant airstream during the presentation of the third (green) circle. A blue circle was presented after the green circle as a cue for a two-alternative forced choice response. Participants responded by button press with the right index and middle finger whether a stimulus was perceived or not. The assignment between response buttons and yes/no choices was counterbalanced across participants. The stimulus duration was 200 ms, the stimulation started 200 ms after inhalation onset and the average ISI amounted to 35.3 (± 8.5) s. Each participant performed four experimental blocks with 40 randomized trials each. Each condition (distilled water, 25%, 50% and 100% propylene glycol) was presented 40 times in total. At the end of the measurement the participants were asked to describe the sensation of the stimuli verbally.

The EEG was recorded from 128 Ag-AgCl electrodes of an active electrode system (EASYCAP, Herrsching, Germany). An electrode on the nose tip served as reference. The electrooculogram (EOG) was monitored with two electrode pairs positioned below and above each eye. The EEG data was digitized with a sampling rate of 1,000 Hz by means of BrainAmp amplifiers (BrainProducts, Munich, Germany). For the offline analysis, the data was filtered with a band-pass of 0.3–100 Hz and downsampled to 500 Hz.

### Data Analysis

Prior to electrophysiological analysis the discrimination performance of the participants was tested by means of d’ analysis (Snodgrass and Corwin, 1988). Offline EEG analysis was performed using Matlab 7.12 (MathWorks, Natick, MA, USA), EEGLAB 11.054b (Delorme and Makeig, 2004) and FieldTrip v.20150318, v.20140801, v. 20140629, v. 20130730, (Oostenveld et al., 2011). EEG artifact rejection was performed in three stages. First, the variance in the data was reduced by removing channels with a high proportion of artifacts. On average data of 122 ± 2 electrodes per participant were analyzed. In the second step, trials containing physiological artifacts such as muscle contraction and electrical artifacts (sudden electrode drifts and jumps) were removed. In the last processing step an independent component analysis (infomax-ICA) was applied to remove artifacts related to eye blinks, eye movements and electrocardiographic activity. After artifact rejection on average 27.9 ± 4.2 trials per condition were maintained: high (mean: 27.5 ± 5.9 trials), medium (mean: 28.3 ± 3.9 trials), low propylene glycol condition (mean: 28.3 ± 4.6 trials), and zero condition (mean: 27.3 ± 5.1 trials). The analysis of the behavioral data was performed by determination of the *d*’ value. The correct detection of trials containing propylene glycol was recorded as a hit. If the participants suspected the presence of propylene glycol during the control condition, the response was logged as a false alarm.

### Chemosensory Event-Related Potential (CSERP) Analysis

For computation of ERPs, the data was low pass filtered at 30 Hz. The continuous EEG recordings were then segmented into 1.5 s data epochs (−500 ms to 1,000 ms around chemosensory stimulus onset). First, EEG data of each participant was averaged separately for all electrode positions. Afterwards, the grand average over all participants was calculated for each condition. The interval between −500 ms to stimulus onset was chosen as baseline. The ERP of the zero condition was subtracted from the ERP of each condition to distinguish the effect of propylene glycol from presentation of distilled water (Supplementary Figures S1, S4). For each condition the individual maximum negative peak was determined in the range of 200–250 ms and the maximum positive peak was calculated in the range of 420–470 ms. Additionally, a two-sided *t*-test was performed for the single comparison between conditions. Bonferroni correction was used to compensate for multiple comparisons. The alpha level for all CSERP comparisons was *p* < 0.05, two-sided.

### Source Reconstruction of the CSERPs

The electrical sources of the CSERP were analyzed at the time range around N1 (200–250 ms) and P2 (420–470 ms) for each condition by means of eLORETA (Pascual-Marqui et al., 2011). The volume conduction model was constructed with a boundary element head model based on the “colin27” template. The BEM model was expressed in MNI coordinates on a 6 mm grid. Labeling of the regions was performed by means of the automated anatomical labeling (AAL) atlas (Tzourio-Mazoyer et al., 2002). For statistical comparisons *t*-tests between each condition containing propylene glycol and the control condition were conducted and cluster permutation statistics was used to control the family-wise error rate (Maris and Oostenveld, 2007).

### Time-Frequency Analysis

In order to analyze spectral changes of oscillatory activity in response to propylene glycol, total power and evoked power were computed separately. Evoked power was obtained by averaging across trials in the time domain and subsequent spectral analysis of the signal. To quantify total power, the frequency transform was computed on single trials and the resulting power values were subsequently averaged. In detail, a 200 ms Hanning window was used to transform each trial into the frequency domain (0.3–30 Hz) and subsequently the data was averaged across trials. Finally, the single participant spectra were baseline corrected (−500 to stimulus onset). The statistical analysis of the conditions containing propylene glycol and the control condition was performed using cluster–based permutation tests (Maris and Oostenveld, 2007) and paired *t*-tests at a significance level of *p* < 0.05.

The evoked power was calculated by averaging the predefined epochs (−500 to 1,000 ms around chemosensory stimulus onset at each electrode) for each condition in the time domain followed by a frequency transformation with a 200 ms Hanning window spanning a range of 0.3–30 Hz. The interval between −500 ms to stimulus onset was chosen as baseline. The statistical analysis of the total power (stimulus vs. baseline) revealed two significant time-frequency clusters (cluster 1: 100–270 ms/7–17 Hz; cluster 2: 400–600 ms/0.3–7 Hz; Figure 4). These time-frequency clusters were also used for the analysis of evoked power.

**Figure 2:**
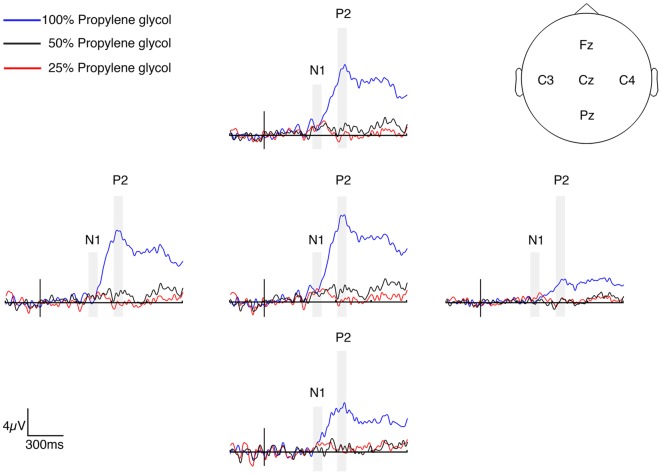
Grand average chemosensory event-related potentials (CSERPs) for three propylene glycol concentration levels. Conditions are plotted as a difference to the control condition to emphasize the effect of the stimulus. The stimulus onset is indicated by a black vertical line. Gray shaded areas indicate the time windows for the analysis of the N1 and P2.

**Figure 3:**
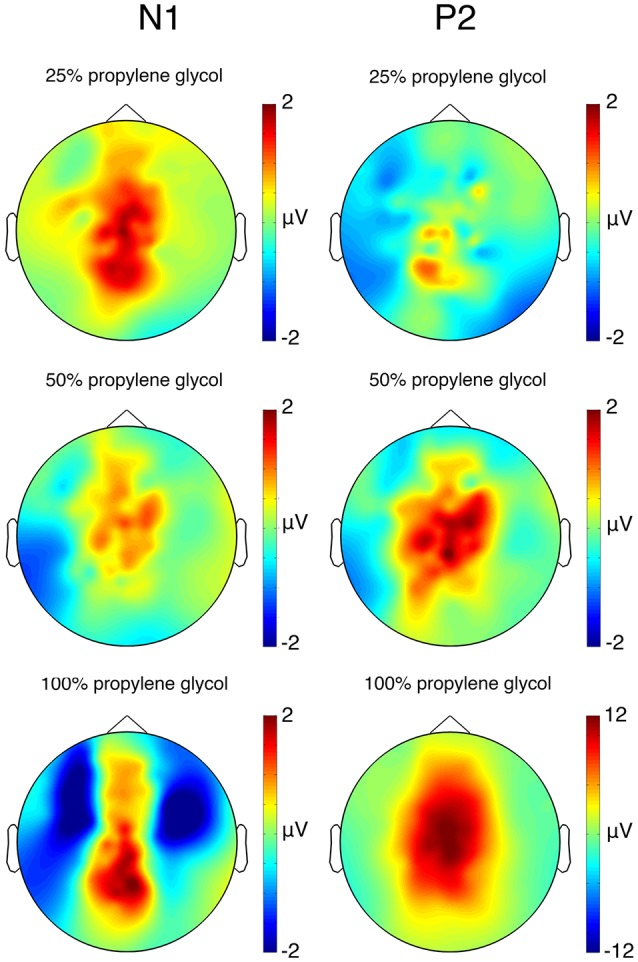
Topography of the CSERP components N1 (200–250 ms) and P2 (420–470 ms) in μV (after subtraction of the control condition). Note that the scaling of the P2 component for 100% propylene glycol is different.

**Figure 4:**
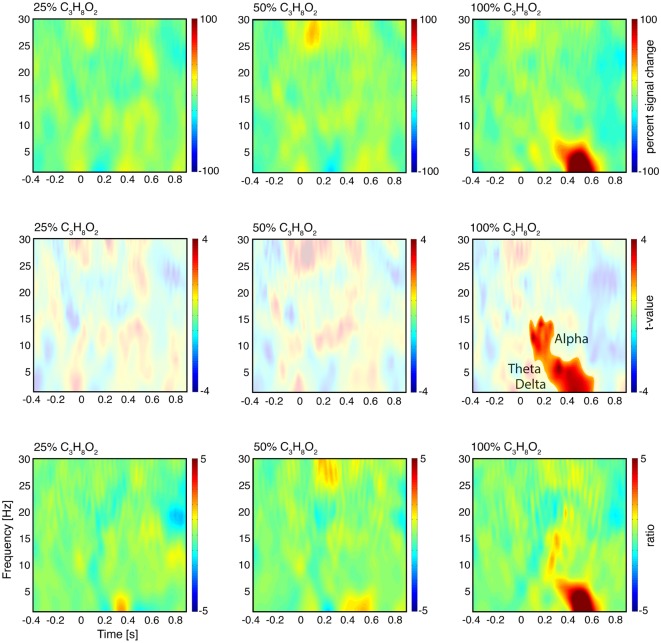
Time-frequency analysis of oscillatory activity. First row: time-frequency representations of total power at electrode Cz after subtraction of the control condition. Stimulus onset is at 0 ms. Second row: significance of total power changes at electrode Cz as revealed by *t*-tests for each condition against the control condition. The highlighted areas are significant (cluster corrected, *p* < 0.05). The colors indicate the *t*-values. Third row: time-frequency representations of evoked power at electrode Cz after subtraction of the control condition.

The spectral analyses of evoked and total power were performed with a Fourier-transformation by means of discrete prolate spheroidal sequences (DPSS, also known as Slepian sequences) taper functions (Mitra and Pesaran, 1999). For both analyses the zero-condition power was subtracted from all conditions to quantify the effect of the nasal stimulation.

### Source Reconstruction of the Time-Frequency Clusters

After the statistical analysis of the total power between the conditions containing propylene glycol and the control condition the electrical sources of the significant time-frequency clusters were analyzed by means of eLORETA (Pascual-Marqui et al., 2011). The analysis was performed for two time-frequency clusters in the 100% propylene glycol condition (cluster 1: 100–270 ms/7–17 Hz; cluster 2: 400–600 ms/0.3–7 Hz). The same volume conduction model and anatomic labeling were used as for source reconstruction of the CSERPs. Prior to statistical analysis the sources were baseline corrected (baseline cluster 1: −400 to −230 ms/7–17 Hz; cluster 2: −400 to −200 ms/0.3–7 Hz). For statistical comparison *t*-tests between the electrical sources of the identified clusters in the 100% propylene glycol condition and the control condition were conducted and cluster permutation statistics was used to control the family-wise error rate (Maris and Oostenveld, 2007).

## Results

### Behavioral Data

The threshold test with propylene glycol revealed an average threshold concentration of 42% ± 28%. In the main experiment, pure propylene glycol was detected with a high probability. In the main experiment, the detection rate decreased with the reduction of the propylene glycol concentration (100% propylene glycol, 96% ± 7.6%; 50% propylene glycol, 60.5% ± 19.5%; 25% propylene glycol, 34.8% ± 14%). The *d*’-values also decreased with the stimulus concentration (100% propylene glycol, 2.7 ± 1; 50% propylene glycol, 1 ± 0.7; 25% propylene glycol, 0.3 ± 0.4). After the measurement 53% of the participants described the sensation as cold, refreshing or similar to the sensation associated with use of toothpaste, 20% felt a burning sensation, 7% reported sweetness, 7% bitterness and 13% were not able to describe the sensation.

### Chemosensory Event-Related Potential (CSERP) Analysis

The results of the *t*-tests comparing the CSERP amplitudes of the conditions containing propylene glycol and the control condition are presented in Table 1. After Bonferroni correction, the significance level was at *p* < 0.003. The statistical analysis of the electrodes Cz, Fz, Pz, C3 and C4 revealed a significant difference of the N1 amplitude between the 50% propylene glycol and the control condition at electrode Pz. Furthermore, a marginally significant difference was observed for the 100% propylene glycol condition (*p* < 0.005). The comparison of the P2 amplitudes revealed significant differences for the 100% concentration condition at electrodes Cz, Pz, Fz, C3 and C4, and over Pz in the 50% condition compared to the control condition (Figure 2). Mean and standard deviation of the latencies of the CSERP components N1 and P2 at electrodes Cz, Fz, Pz, C3 and C4 are shown in Table 2. The N1 and P2 latencies did not show any significant differences between the propylene glycol conditions. Figure 3 displays the topographies of the N1 and P2 CSERP components (see Supplementary Figures S2, S3for ERPs of all electrodes and topographies of all conditions).

**Table 1:** Results of *t*-tests comparing chemosensory event-related potential (CSERP) amplitudes of the conditions containing propylene glycol (100%, 50% and 25%) against the control condition at time windows of N1 and P2 at electrodes Cz, Fz, Pz, C3 and C4.

N1					
C_3_H_8_O_2_	Pz	Cz	Fz	C3	C4
100%	*p* = 0.005	*p* = 0.29	*p* = 0.92	*p* = 0.25	*p* = 0.82
	*t* = 3.27	*t* = 1.08	*t* = 0.09	*t* = −1.1	*t* = 0.22
50%	*p* = 0.001	*p* = 0.06	*p* = 0.93	*p* = 0.59	*p* = 0.08
	*t* = 3.87*	*t* = 1.98	*t* = −0.08	*t* = 0.54	*t* = 1.88
25%	*p* = 0.005	*p* = 0.09	*p* = 0.83	*p* = 0.49	*p* = 0.34
	*t* = 3.24	*t* = 1.78	*t* = 0.21	*t* = 0.69	*t* = 0.98
**P2**					
**C_3_H_8_O_2_**	**Pz**	**Cz**	**Fz**	**C3**	**C4**
100%	*p* < 0.001	*p* < 0.001	*p* < 0.001	*p* < 0.001	*p* < 0.001
	*t* = 6.6*	*t* = 6.21*	*t* = 5.09*	*t* = 5.73*	*t* = 5.3*
50%	*p* = 0.001	*p* = 0.02	*p* = 0.52	*p* = 0.05	*p* = 0.03
	*t* = 3.98*	*t* = 2.51	*t* = 0.65	*t* = 2.09	*t* = 2.32
25%	*p* = 0.01	*p* = 0.14	*p* = 0.9	*p* = 0.23	*p* = 0.09
	*t* = 2.89	*t* = 1.54	*t* = 0.12	*t* = 1.25	*t* = 1.79

The significant t-values are labeled with a *. The significance level after Bonferroni correction was at p < 0.003.

**Table 2:** Mean and standard deviations of the grand average CSERP component latencies (N1 and P2) at electrode Cz, Fz, Pz, C3 and C4.

N1					
C_3_H_8_O_2_	Pz	Cz	Fz	C3	C4
100%	233 ± 20	232 ± 17	235 ± 12	231 ± 15	236 ± 11
50%	224 ± 19	227 ± 18	226 ± 17	225 ± 19	227 ± 16
25%	226 ± 19	224 ± 17	226 ± 15	228 ± 16	230 ± 22
**P2**					
**C_3_H_8_O_2_**	**Pz**	**Cz**	**Fz**	**C3**	**C4**
100%	441 ± 19	447 ± 17	446 ± 19	443 ± 18	442 ± 19
50%	445 ± 16	445 ± 19	446 ± 18	442 ± 19	439 ± 12
25%	433 ± 12	436 ± 17	449 ± 16	441 ± 15	440 ± 17

*The values are shown in milliseconds*.

### Time-Frequency Analysis

**Figure 5:**
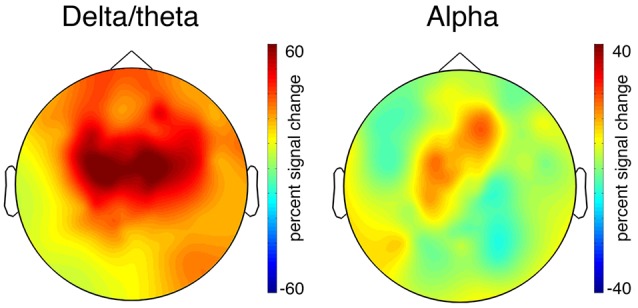
Scalp distribution of the significant total power time-frequency clusters (see Figure 4, second row, third column). The colors indicate the power increase in Delta/theta: 100% propylene glycol minus the control condition (260–620 ms, 0.3–7 Hz). Alpha: 100% propylene glycol minus the control condition (100–240 ms, 8–15 Hz). Note that the topographies have a different scaling.

A cluster-based permutation test (*p* < 0.05) of the oscillatory activity of the 100% propylene glycol condition and the control condition revealed low frequency power increases (*p* = 0.004; Figure 4, second row, third column). Delta/theta power (0.3–7 Hz) increased in the time range of 260–620 ms and alpha power (8–15 Hz) with a latency of 100–240 ms. The scalp topography revealed a maximum power increase of delta and theta at the scalp vertex (Figure 5), while the peak of alpha power was measured at C4 and central-frontal electrodes. The 25% and 50% propylene glycol conditions did not show any significant power modulation. The same statistical tests were performed for all significant clusters in the evoked power. Only the delta/theta cluster differed significantly from the control condition (*p* = 0.003).

### Source Reconstruction of the CSERPs

**Figure 6:**
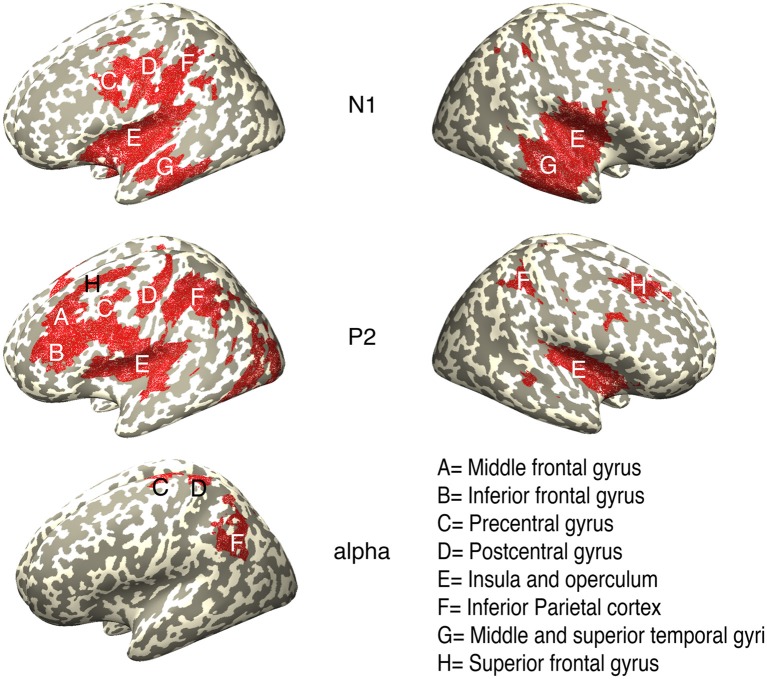
Sources of the CSERP components N1 (200–250 ms), P2 (420–470 ms) and the time-frequency cluster in the alpha-band (100–240 ms/8–15 Hz) in response to pure propylene glycol stimulation analyzed by means of exact low-resolution electromagnetic tomography (eLORETA). Highlighted regions are significant clusters revealed by a cluster permutation test (*p* < 0.025).

The statistical analysis in the source space with a cluster permutation test (*p* < 0.025) at 200–250 ms (N1) and 420–470 ms (P2) revealed significant clusters for the 100% propylene glycol condition compared to the control condition (Figure 6). Brain regions such as the postcentral gyrus, the insula and adjacent operculum, the thalamus and the cerebellum could be identified during time points for N1 and P2. Except for the postcentral gyrus, which was activated contralaterally to the stimulation site at N1 and bilaterally at P2, all mentioned regions were activated in both hemispheres during N1 and P2. Similar to the postcentral gyrus, the caudate nucleus and middle frontal gyrus showed contralateral activity during N1 and bilateral activity during P2. In contrast the analysis revealed a bilateral activation of the superior temporal gyrus in the time range of N1 and a contralateral activation during P2. The anterior part of the cingulate cortex (ACC) was only active during P2 (contralateral). Additionally, the analysis revealed bilateral activation of the post (PCC) and mid cingulate cortex (MCC) during N1 and P2. The analysis of the other conditions (50% and 25% propylene glycol) did not reveal any significantly activated regions.

### Source Reconstruction of the Time-Frequency Cluster in the Alpha-Band

The statistical analysis of the electrical sources of the time-frequency cluster in the alpha range (100–270 ms/7–17 Hz) between the 100% propylene glycol condition and the control condition revealed contralateral activation in the parietal areas, like the inferior- and superior parietal lobe, and the postcentral gyrus. Additionally, significant activation in the contralateral precentral gyrus and ipsilateral activation of the cerebellum was found.

## Discussion

The aim of this study was to investigate whether nasal propylene glycol stimulation, often used to dilute fragrances as a putative neutral substance, elicits subjective perceptions and brain responses that would account for activity originating from olfactory or trigeminal inputs or a combination of both sensory systems. We measured detection thresholds and analyzed the neural correlates of propylene glycol stimulation using multi-channel EEG. We showed that ERPs were modulated in amplitude depending on the concentration of the substance. Participants reported that propylene glycol generates a cooling sensation in the nasal cavity. Threshold tests revealed that, contrary to the general assumption, participants perceive propylene glycol already at concentrations which are often used for dilution of pure olfactory substances. Therefore, use of propylene glycol as a diluent can confound results and render interpretation difficult in studies where solely olfactory stimulation is intended.

Stimulants such as PEA or vanillin, which are supposed to act mainly on olfactory receptors in low concentrations, allow to study the olfactory sense specifically (Doty et al., 1978). However, for the examination of stimulus-response functions, the manipulation of the stimulus concentration requires the dilution of the fragrance, which can be accomplished in two ways. The first possibility is flow-dilution. A neutral dilution airstream is passed to the airstream containing the fragrance. The disadvantage of this method is that the air currents must reach a minimum pressure to generate a fast rising of the stimulus concentration. A slow increase of the concentration would lead to a habituation effect and would not produce clear neural responses. Airstreams of current flow-olfactometers must reach a minimum air pressure of 0.5 l/min. The total flow (C) is composed of the airstreams for the fragrance (O) and the dilution (D; C = D + O). The common air pressure, which is applied for a well tolerated measurement without additional irritation of the mucosa, is about 8 l/min. Thus, the stimulus can be diluted down to about 6% by adding a dilutive flow of 7.5 l/min. Depending on the stimulus substance, the average olfactory detection threshold of healthy individuals often lies below this value. Behavioral measurements with the Sniffin’ Sticks (n-butanol) found a threshold below 1%. (Hummel et al., 2007). Since the olfactometer applies the substance directly into the nostrils, it is to be expected that using the olfactometer the threshold would be even lower. The stimulation close to the threshold can thus be achieved only by static dilution of the fragrances. However, substances such as PEA are not water-soluble. Therefore, they are often diluted with propylene glycol. However, its consideration as a neutral solvent is clearly challenged by both detection thresholds and ERPs recorded in healthy participants in the present study.

The analysis of the behavioral and electrophysiological data suggests a trigeminal effect of propylene glycol although an additional olfactory involvement cannot be excluded, because trigeminal and olfactory responses are very similar at late latencies beyond 300 ms in response to volatile stimuli (Iannilli et al., 2013). Elicitation of responses following non-odorant nociceptive CO_2_ stimuli at the absence of responses following odorant stimuli can serve to objectively document disturbed olfaction (Hummel and Kobal, 1992). In addition to their dissociation by distinct olfactory lesions, stronger P2 at electrodes contralateral to the stimulated nostril suggests a trigeminal mediation (Hummel and Kobal, 1992). Stimulation with a high concentration of propylene glycol elicits a P2 component at central electrodes in our study in which we only stimulated the right nostril. Although the trigeminal or olfactory origin of responses in our study remain unclear, we observed a correspondence between the stimulus concentration, the detection rate and the P2 amplitude in the CSERPs. Reducing the concentration to 50% resulted in a lower detection rate and a reduction of the P2 amplitude, which only differed from the control condition at electrode Pz. The analysis of the weakest concentration (25%) showed the lowest detection rate. In addition, the P2 amplitude did not differ at any electrode from the control condition. Since the threshold test revealed an average propylene glycol detection threshold of 42 ± 28%, it can be assumed that the 25% condition is sub-threshold. The CSERP analysis showed that relative to the baseline there is neither a clear N1 peak nor do any of the propylene glycol conditions differ significantly from the control condition in that time range. Typical parameters, which have an effect on CSERP amplitudes, are the age of the participants, stimulus concentration, length of the ISI, the respiration cycle, or attention (Geisler and Murphy, 2000; Stuck et al., 2006; Bensafi et al., 2008; Kassab et al., 2009). Since the average age of the participants was low in this study (23.7 ± 3.5 years), the stimulus duration and the ISI met the standards, and in all conditions attention was directed towards the stimulus, it can be assumed that the crucial factor is the stimulus itself. Further tests might reveal whether nasal propylene glycol stimulation elicits a clear N1 component. The comparison of the P2 components at the contralateral and ipsilateral electrodes could be suitable to identify the type of chemosensory stimuli, but should be supplemented with further analysis methods.

Huart et al. (2012) analyzed phased-locked oscillatory activity after trigeminal stimulation and found an increase of power in the delta/theta-band, which is similar to the results of this study. The same analysis after olfactory stimulation did not show any significant results. One explanation could be that the delta/theta power increase is related to the CSERP. Since olfactory ERPs are not as pronounced as trigeminal ERPs, they were not clearly visible in the frequency domain (Iannilli et al., 2013). In addition, the time-frequency analysis in this study revealed an early phased-locked alpha power increase, which might be attributable to the inspiration (Masaoka et al., 2005).

The results of the source reconstruction complemented the findings of the CSERP analysis. The source analysis of the neural responses to 100% propylene glycol for the time windows of the CSERP components N1 and P2 revealed activation of regions, which are known to be involved in processing of olfactory and trigeminal stimuli, such as the insula and adjacent operculum, the thalamus, or the cerebellum in both hemispheres (Hummel et al., 2005; Boyle et al., 2007b; Iannilli et al., 2007; Bensafi et al., 2008). Iannilli et al. (2008) already showed in an EEG study activation of the insula after chemical, mechanical, and electrical trigeminal stimulation and found that the combination of stimuli affects the side of activation.

In a meta-analysis of human functional brain imaging data, 15 studies with chemosensory stimuli (CO_2_) were evaluated and surprisingly the largest cluster containing significant ALE scores (Assembly Likelihood Evaluation) was not as expected in the somatosensory cortex, but in the frontal operculum and the insular cortex (Albrecht et al., 2010). There appears to be a relationship between the processing of chemosensory stimuli and the insular and adjacent operculum. Since both are activated for painful and non-nociceptive stimuli, they might generally be higher integrative regions for processing of trigeminal stimuli (Treede et al., 1999).

The activation of the cerebellum with chemosensory stimuli is not well understood. It has been proposed that this might relate to the regulation of respiration. Early observations suggested that the cerebellum regulates the sniff volume (Sobel et al., 1998a,b, 2000). However, neuroimaging studies showed that the cerebellum is also activated during passive stimulation with oral breathing (Yousem et al., 1997). Regardless of the type of breathing the cerebellum seems to be involved in breathing regulation. A study comparing olfactory and trigeminal stimulation revealed that the cerebellar activation is indeed present in both, but greater after trigeminal stimulation (Iannilli et al., 2013). This may be related to the fact that CO_2_ stimulation causes apnea, which explains a stronger activation of the areas involved in breathing regulation (Boushey and Richardson, 1973; Alvaro et al., 1992; Yavari et al., 1996). The nociceptive effect of CO_2_ might also account for the cerebellar response, which is also observed following painful and aversive heat stimuli (Casey et al., 2001).

Chemosensory stimulation with propylene glycol also activated the cingulate cortex. The mid-cingulate and posterior cingulate cortex were active in both hemispheres during N1 and P2. During P2 anterior cingulate cortex was also activated on the left hemisphere (contralateral to the stimulus side). A connection between chemosensory stimulation and the cingulate cortex has also been observed in other studies (Savic et al., 2002; Boyle et al., 2007a, b; Iannilli et al., 2007). An fMRI study showed that anterior cingulate regions are related to stimulus awareness, cognitive processing and basic sensory processing (Büchel et al., 2002). Another fMRI study, in which high and low concentrations of CO_2_ were compared, showed that mid-cingulate and posterior cingulate play a crucial role in the encoding of stimulus concentration (Bensafi et al., 2008).

The source analysis also revealed a contralateral activation of the caudate nucleus during N1 and a bilateral activation during the P2 interval. Earlier studies investigating olfactory stimulus processing found a relation between the caudate nucleus and olfactory stimulation (Savic et al., 2000; Hummel et al., 2005; Iannilli et al., 2013). The function of the caudate was attributed to the discrimination of odor quality (Savic et al., 2000; Hummel et al., 2005). However, as an activation of the caudate nucleus was also observed after trigeminal stimulation with CO_2_, it likely is involved in other processes that are not purely olfactory (Iannilli et al., 2008; Hummel et al., 2009).

Two further regions, which in our data were found to be significantly activated compared to the control condition, are the superior temporal and middle frontal gyrus. An activation of these regions after trigeminal stimulation has already been reported in a meta-analysis of brain imaging data (Albrecht et al., 2010). Generally, these regions appear to play a role in associative processing of chemosensory stimuli. Cerf-Ducastel and Murphy (2006) investigated olfactory recognition memory and found a correlation of the middle frontal gyrus and the increasing familiarity to a fragrance (Cerf-Ducastel and Murphy, 2006). Moreover, it was already shown that the middle frontal gyrus plays a crucial role in odor evaluation and discrimination (Porter et al., 2005; Bensafi et al., 2008). The superior temporal sulcus was found to be involved in the perception of olfactory and trigeminal stimuli and is even associated with emotional processing and the localization of fragrances (Kettenmann et al., 1996; Royet et al., 2000; Calvert and Thesen, 2004; Hummel et al., 2005). In addition it should be emphasized that the superior temporal sulcus is known as a multisensory region and is therefore likely to be involved in trigeminal and olfactory processing (Kettenmann et al., 1996; Hummel et al., 2005).

The postcentral gyrus was the only area, which was not activated in both hemispheres during the N1 interval, but was contralaterally activated in the left hemisphere (200–250 ms). The contralateral activation pattern (right nostril was stimulated) is an indicator for a trigeminal stimulus response (Iannilli et al., 2007).

The measurement of the detection thresholds revealed that healthy participants can detect propylene glycol. Furthermore, the higher contralateral P2 amplitudes and the observation of activity in the contralateral postcentral gyrus suggest a trigeminal effect. Whether the percept is based solely on the trigeminal pathway or involves also the olfactory tract cannot be distinguished unambiguously based on the present data, but could be investigated by presenting propylene glycol to anosmic patients (Doty et al., 1978). Nevertheless, based on our results the usefulness of propylene glycol for the dilution of fragrances is at least questionable.

As a potential alternative to propylene glycol for the dilution of fragrances, dipropylenglycole could be tested. This substance has properties similar to propylene glycol and is frequently used as a solvent for cosmetics. In several studies dipropylenglycole was already applied for diluting fragrances (Kempter, 2008; Sorokowska et al., 2017). Threshold tests and electrophysiological studies with dipropylenglycole could provide evidence on whether this substance would be suited for diluting fragrances like PEA or vanillin.

In conclusion, our study reveals that high concentrations of propylene glycol create a cooling sensation and demonstrated neural responses suggesting a trigeminal effect. Therefore, we suggest that propylene glycol should not be used for dilution of olfactory fragrances in cases where pure olfactory responses are targeted by the investigation.

## Data Availability

The raw data supporting the conclusions of this manuscript will be made available by the authors, without undue reservation, to any qualified researcher.

## Author Contributions

MS, NS, TS, AE and JL contributed to the conception and design of the study. NS and MS performed the experiment. MS, NS, TS, and UF performed the analysis of the data. The manuscript was written by MS, NS, and TS. The manuscript has been edited and approved by all the authors.

## Conflict of Interest Statement

The authors declare that the research was conducted in the absence of any commercial or financial relationships that could be construed as a potential conflict of interest.
