# Supplementary material for: Chemosensory Event-Related Potentials in Response to Nasal Propylene Glycol Stimulation
Source: Front Hum Neurosci. 2019 Mar 20;13:99. doi: 10.3389/fnhum.2019.00099 (PMC6435593; doi:10.3389/fnhum.2019.00099)
Supplement: Supplementary file 1 [file Data_Sheet_1.pdf]

Chemosensory event-related potentials in response to nasal propylene glycol stimulation

Mohammad Sirous[1*], Nico Sinning[1*], Till R. Schneider[1], Uwe Friese[1,3], Jürgen Lorenz[2], Andreas K. Engel[1]

[1] Department of Neurophysiology and Pathophysiology, University Medical Center Hamburg-Eppendorf, 20246 Hamburg, Germany

[2] Faculty of Life Science, Laboratory of Human Biology and Physiology, Applied Science University, 21033 Hamburg, Germany

[3] Faculty of Life Sciences, MSH Medical School Hamburg, 20457 Hamburg, Germany

* Both authors contributed equally to this manuscript

Correspondence should be addressed to:
Dr. Mohammad Sirous
Dept. of Neurophysiology and Pathophysiology
University Medical Center Hamburg-Eppendorf
20246 Hamburg
Dr.mohammadsirous@gmail.com

Supplementary material

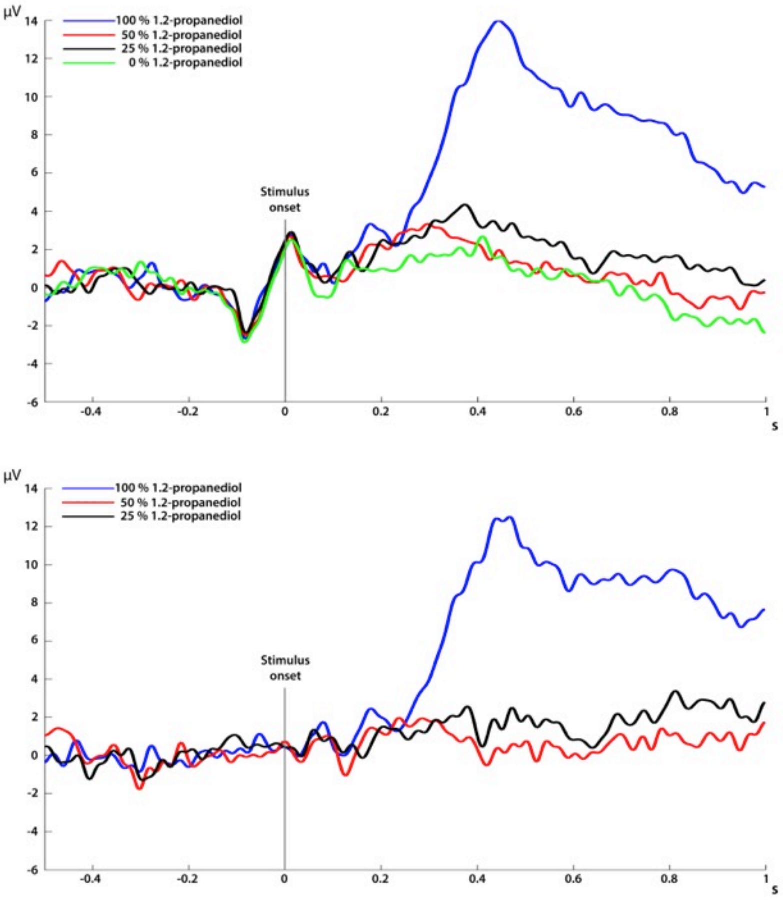

Supplementary Figure 1: Baseline corrected (-500 – 0 ms) evoked potentials. The x-axis shows the time in ms and the y-axis displays the power in μ V.

Top: CSERPs of all conditions at electrode Cz. Bottom: CSERPs of the conditions containing propylene glycol after subtraction of the control condition (electrode Cz). The ERPs in the top panel include a visual evoked potential during the stimulus onset, which was produced by the stimulus cue. Subtraction of the control condition reveals the pure response to the chemosensory stimulus (bottom panel).

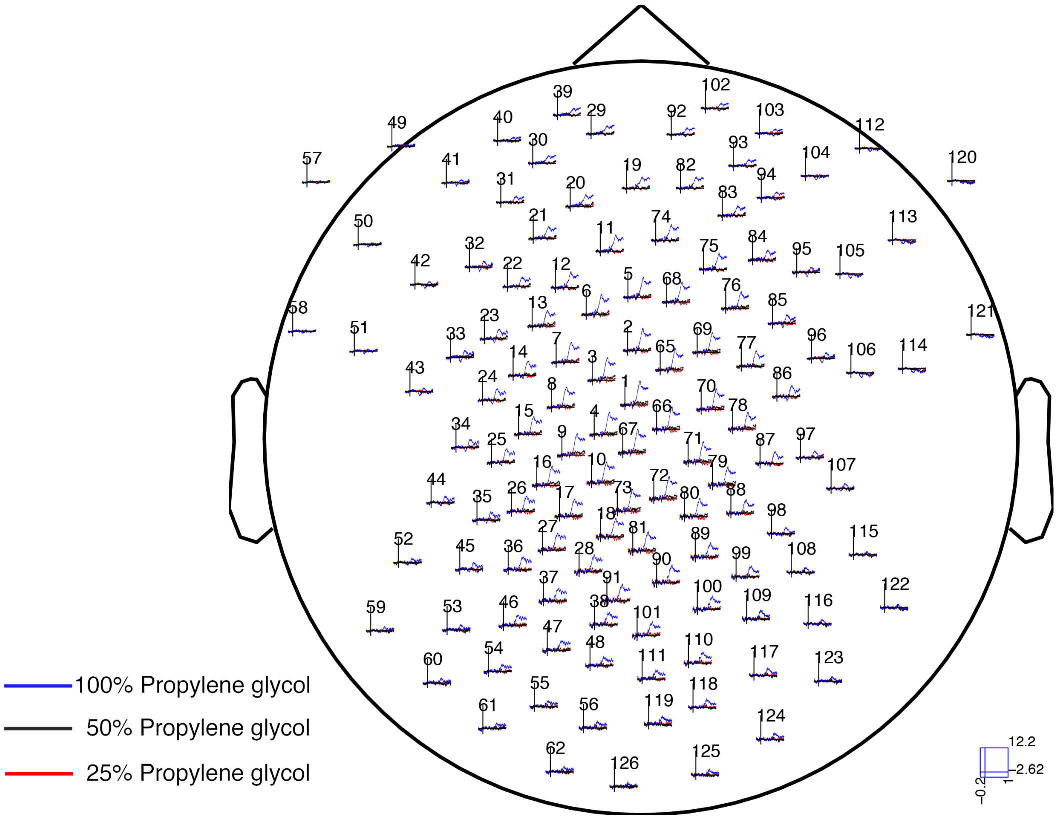

Supplementary Figure 2: Grandaverage CSERPs at all electrodes (baseline corrected (-500 – 0ms)). The x-axis shows the time in ms and the y-axis displays the power in μV. All conditions containing propylene glycol are plotted after subtraction of the control condition.

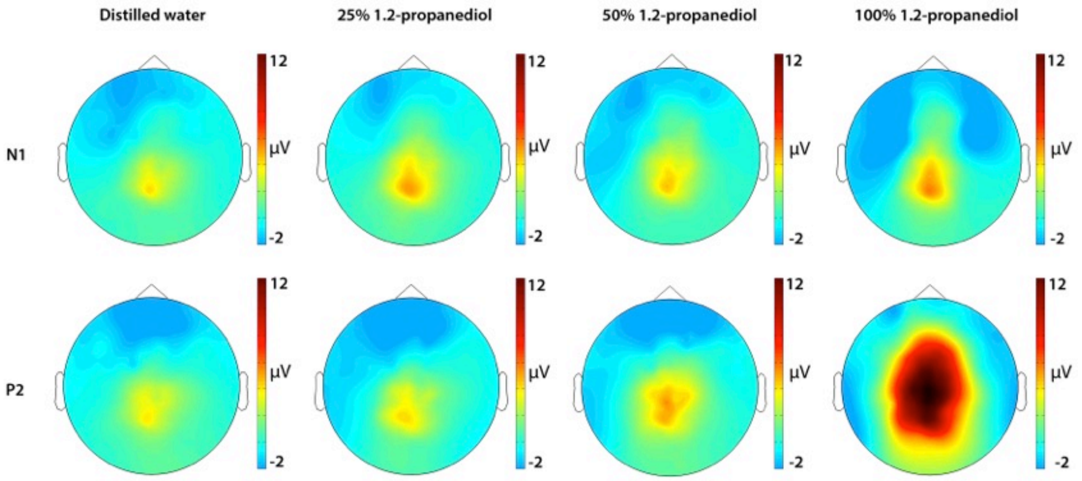

Supplementary Figure 3: Topography of EEG responses at N1 (240ms) and P2 (450ms) in µV for all conditions.

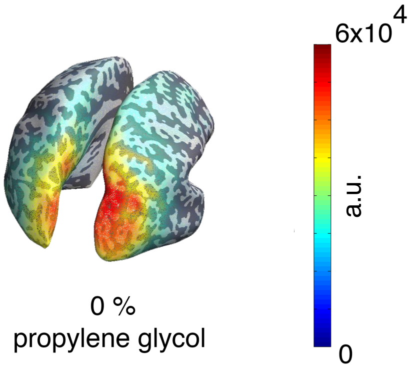

Supplementary Figure 4: Source analysis of the visual evoked potential (125 ms after colour change, see supplementary figure 1) in the control condition by means of eLORETA. The analysis reveals occipital activation. The visual response seems to be related to the colour change of the circle before chemosensory stimulus onset, which slightly changed the luminance.
